# We did it their way, flexible and robust patient recruitment to the IQuaD trial

**DOI:** 10.1186/1745-6215-14-S1-P81

**Published:** 2013-11-29

**Authors:** Anne Duncan, Thomas Lamont, Jan Clarkson, Craig Ramsay

**Affiliations:** 1 University of Aberdeen, Aberdeen, UK; 2 University of Dundee, Dundee, UK

## 

The NIHR HTA funded IQuaD (Improving the Quality of Dentistry)Trial is a 5 year multi-centre, cluster randomised, open trial with blinded outcome evaluation based in dental primary care in Scotland and the North East of England. It compares scale and polish intervals and oral hygiene advice delivery for the maintenance of oral health. We aim to recruit 1860 adults across 60 dental practices. Trials of this size have not previously been carried out in primary dental care, reflecting these uncertainties Outcome Assessors (qualified Hygienists and Dental Nurses) are employed by the trial to go into dental Practices to consent eligible patients as well as collecting baseline measurements.

Novel strategies used include:

• 1. Trial staff arranging and managing clinical recruitment sessions with Practice staff in advance

• 2. Inviting patients to attend recruitment sessions in line with each participating Practices' routine methods of contacting patients

• 3. Sending information about the Trial along with an invitation letter and baseline questionnaire to patients in advance of clinical recruitment sessions

• 4. Organising flexible appointment systems with Practices allowing patients to opt out of the Trial in advance of their dental appointment or following discussion with trial and dental staff at recruitment sessions

Based on our experiences in IQuaD, we will discuss the challenges of incorporating trial recruitment processes into general dental primary care and how these strategies enabled us to accurately predict and maximise recruitment to meet targets.

